# The presentation of spontaneous splenic rupture in a COVID-19 patient: a case report

**DOI:** 10.1186/s12893-020-00887-5

**Published:** 2020-10-02

**Authors:** Mohammadreza Mobayen, Saeed Yousefi, Mohammadsadegh Mousavi, Amin Shafighi Anbaran

**Affiliations:** Department of General Surgery, Guilan University of Medical Sciences, Rasht, Iran

**Keywords:** Case report, Surgery, Spleen, COVID-19

## Abstract

**Background:**

Splenic rupture is an emergency condition and a vast number of cases are secondary to trauma. Several underlying pathologies have also been associated with splenic rupture, such as hematological diseases, malignancies, and infectious and inflammatory diseases.

**Case presentation:**

The patient was a 52-year-old man who referred to the Poursina Hospital in Rasht while complaining of abdominal pain from the day before hospitalization. The patient reported a history of lethargy, fever, and nausea. In the examinations performed, there was a brief tenderness in the patient’s epigastrium. The patient was monitored and about 12 h after hospitalization, ill appearance, respiratory (respiratory distress) symptoms, and high fever were reported for the patient. According to the examination, the patient was immediately transferred to the operating room and underwent laparotomy. During the operation, contrary to our expectations, a lot of blood (about 1000 cc) was observed in the patient’s abdomen. After blood suctioning, the left upper quadrant (LUQ) was bleeding and the rupture of the spleen could also be observed. Therefore, a splenectomy was performed. In the examinations performed for the patient, the patient’s rtPCR test confirmed COVID-19.

**Conclusion:**

The evaluation of the spontaneous splenic rupture (SSR) in our case shows that this type of risk should also be considered in patients with COVID-19 who refer to medical centers with abdominal pain, and if more cases are reported, the correctness of this process can be commented on.

## Background

Splenic rupture is an emergency condition and a vast number of cases are secondary to trauma. Several underlying pathologies have also been associated with splenic rupture, such as hematological diseases, malignancies, and infectious and inflammatory diseases [1–3]. Atraumatic splenic rupture (ASR) is rare [1, 2]. Splenic rupture is not considered in the differential diagnosis of abdominal pain in the absence of trauma, the results of which may be catastrophic [4].

The diagnosis of splenic rupture is a clinical finding confirmed by either Computed tomography scan (CT scan) or laparotomy (in hemodynamically unstable patients). Several grading systems have been established for splenic rupture based on CT scan or ultrasound findings and each has been found to be effective in contributing to management and decision making [4].

However, there has been a tremendous amount of case reports of atraumatic splenic rupture, there are not any comprehensive assessment to reveal the incidence rate, causes, specific symptoms, available management methods, and the prognosis [3].

## Case presentation

The patient was a 52-year-old man who referred to the Poursina Hospital in Rasht while complaining of abdominal pain from the day before hospitalization. The patient reported a history of tiredness, fever, and nausea. In the examinations performed, there was a brief tenderness in the patient’s epigastrium. The patient’s initial pressure was 80.120 with a pulse rate of 95. The patient’s fever was not detected upon arrival. For the patient, an upright abdominal radiograph, abdominal and pelvic ultrasounds, and blood tests including CBC,BUN,Cr,PT,PTT,INR,LFT,Amylase,Lipase,LDH were requested.

**Fig. 1:**
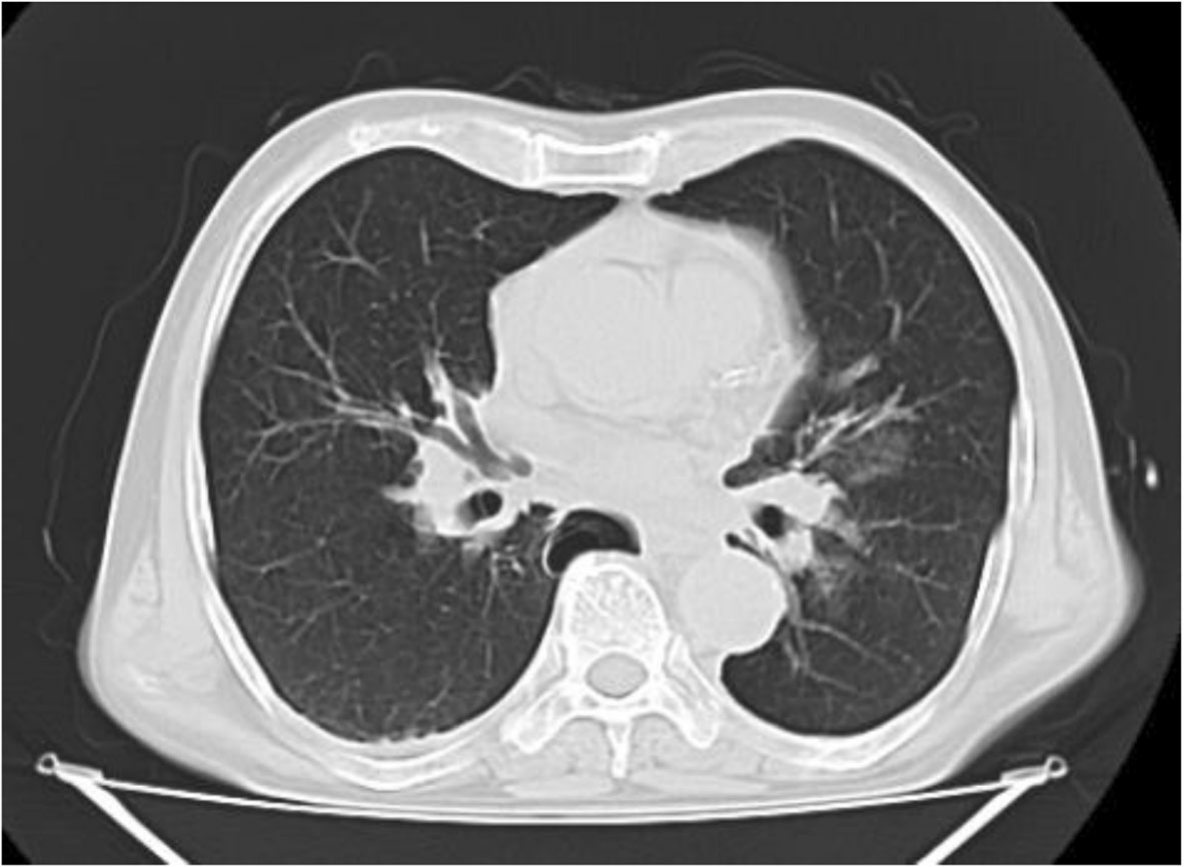
Primary lung CT scan. No pathological lesion was observed on upon arrival lung CT scan of the patient

**Fig. 2:**
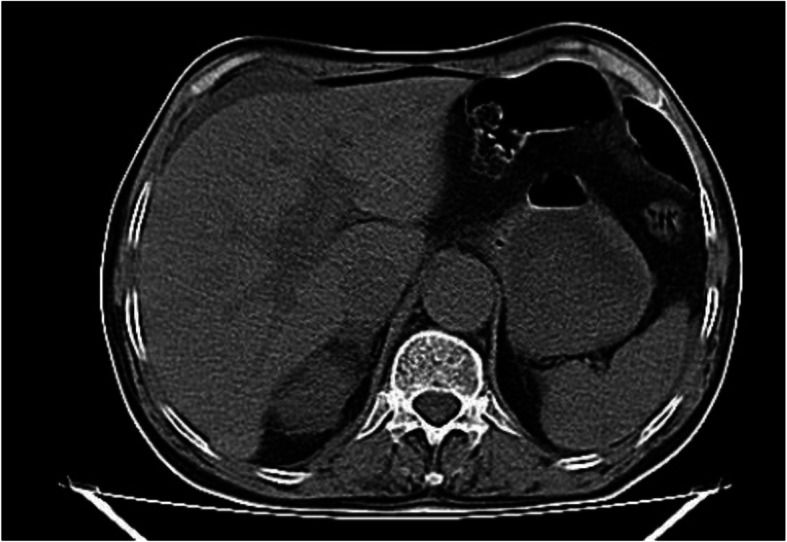
Abdomen view in Primary CT scan. A small amount of perihepatic and perisplenic fluid was observed on the patient’s CT scan

The patient’s upright abdominal radiograph was normal. Perihepatic and perisplenic fluid and gallstones were seen on the patient’s ultrasound. On laboratory findings, White Blood Cells (WBC) = 4000 /μL with 30% lymphocytes were reported. Hemoglobin (Hb) = 11 g/dL and Platelets (PLT) = 245,000 /μL were found. Other findings were normal. No pathological lesion was observed on the patient’s lung CT scan (Fig. 1). A small amount of perihepatic and perisplenic fluid was observed on the patient’s CT scan (Fig. 2).

The patient was monitored and about 12 h after hospitalization, ill appearance, respiratory (respiratory distress) symptoms, and high fever were reported for the patient. The patient’s O2Sat was 96%. Due to the exacerbation of the patient’s respiratory symptoms, the CT scan of the lungs was requested again for the patient and the tests were repeated for suspicion of coronavirus.

**Fig. 3:**
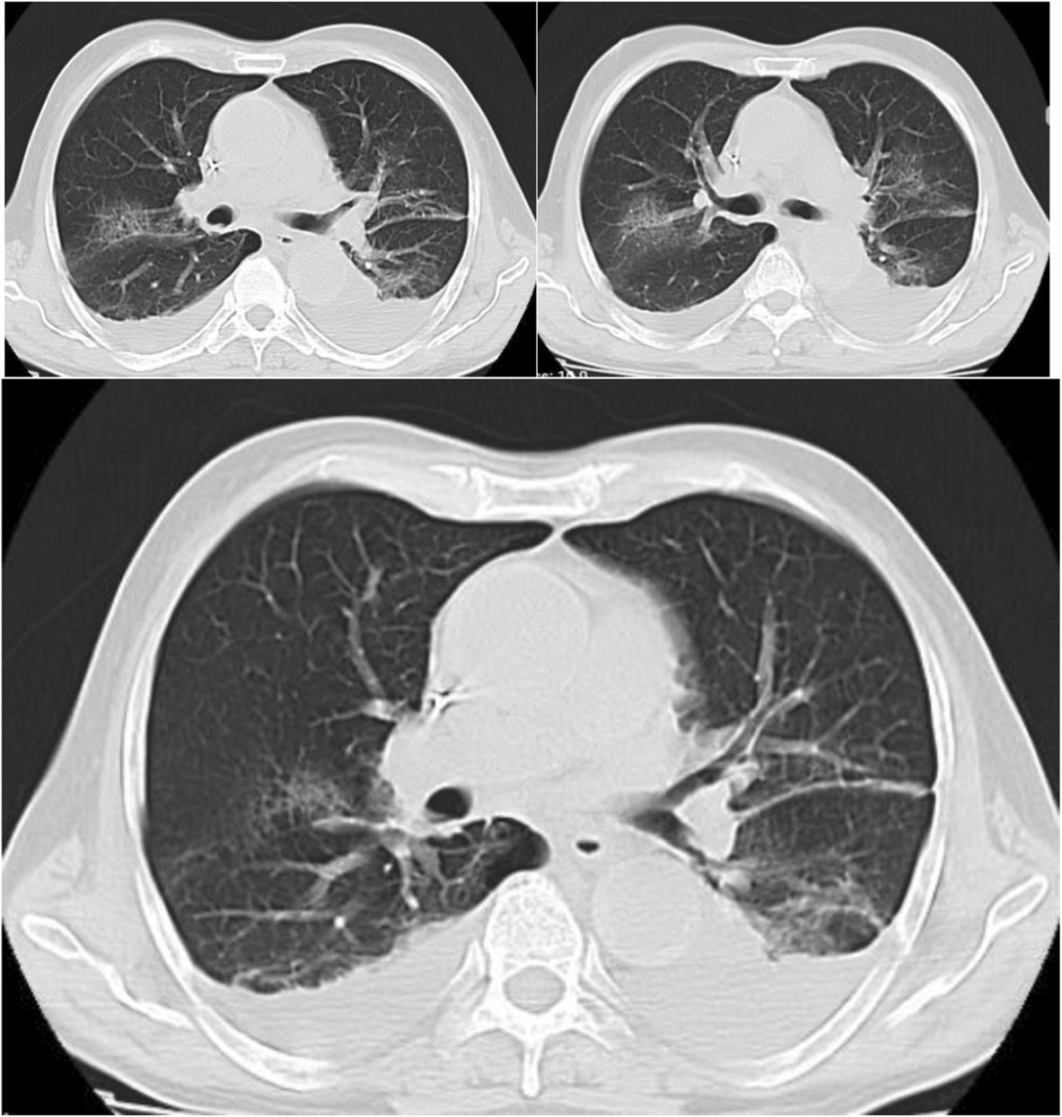
Secondary lung CT scan. The second CT scan was performed about 18 h after the initial examination. Patchy ground-glass lesions along with bilateral pleural effusion were seen on the CT scan of the patient’s lungs

**Fig. 4:**
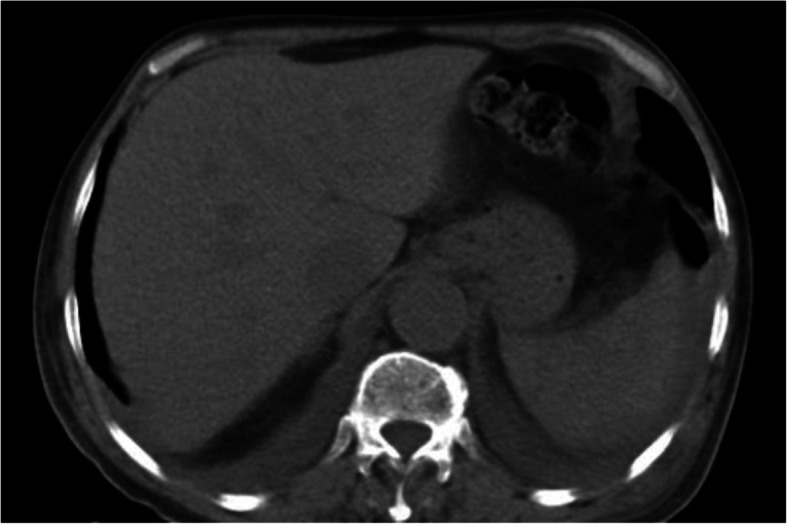
Abdomen view in Secondary CT scan. An extensive fluid collection around the spleen was observed on the CT scan performed for the patient

**Fig. 5:**
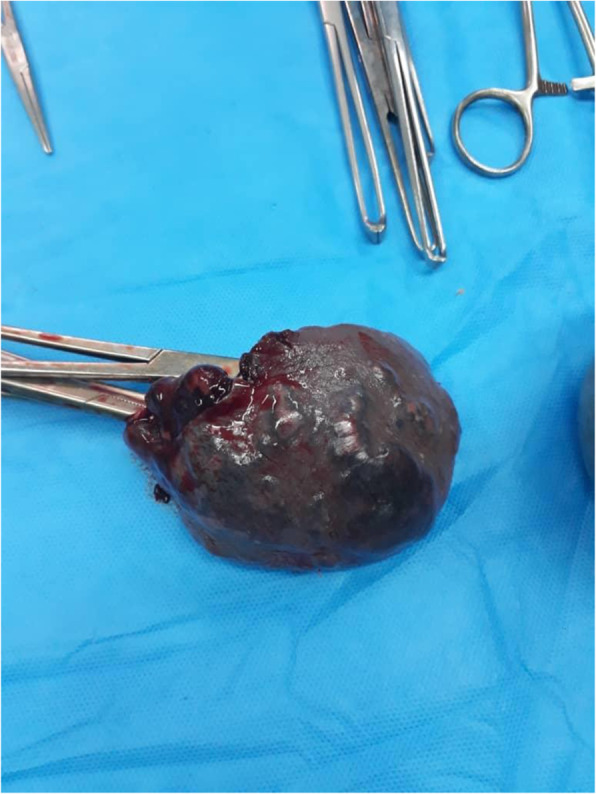
Appearance of the spleen after splenectomy. The rupture of the spleen could also be observed. Therefore, a splenectomy was performed

The second CT scan was performed about 18 h after the initial examination. Patchy ground-glass lesions along with bilateral pleural effusion were seen on the CT scan of the patient’s lungs (Fig. 3). A bilateral chest tube was inserted for the patient according to the volume of the fluid, and according to the patient’s CT scan and respiratory symptoms, he was transferred to the specialized COVID-19 unit. In the new blood tests, Hb = 9.5 g/dL, WBC = 4800/μL, and PLT = 195,000 /μL were observed. An extensive fluid collection around the spleen was observed on the CT scan performed for the patient (Fig. 4). The examination of the patient’s abdomen changed significantly about 20 h after the initial examination and became generalized rebound tenderness. However, the patient’s blood pressure was still 70.110 and the pulse rate was 90. According to the examination, the patient was immediately transferred to the operating room and underwent laparotomy. During the operation, contrary to our expectations, a lot of blood (about 1000 cc) was observed in the patient’s abdomen. After blood suctioning, the left upper quadrant was bleeding and the rupture of the spleen could also be observed. Therefore, a splenectomy was performed (Fig. 5).

In the examinations performed for the patient, the patient’s PCR confirmed COVID-19. The patient was admitted to the ICU after surgery, and within 3 days after surgery, the patient was extubated. The patient’s chest tubes were removed within 1 week after the surgery according to the radiographs performed. After a concomitant treatment for COVID-19 and during a two-week hospital stay, the patient was discharged in good general condition.

During the examinations performed and the history taken several times from the patient and the patient’s companion, no history of the trauma was given and the changes in the patient’s CT scan and the patient’s examinations occurred very quickly.

The LE cell, ANA, and HIV as well as the Monospot, RF and Wright agglutination test were negative. The peripheral blood smear and the Widal agglutination test were negative for malaria and VCA Antibody IgM and IgG were negative for Epstein - Barr virus (EBV).

In the macroscopic study of splenic pathology, a spleen tissue with dimensions of 11× 8 × 5 cm and a weight of 350 g and a laceration with a size of 2 × 2 cm were observed and in the microscopic study, a focally hemorrhagic area was reported.

## Discussion and conclusions

Coronaviruses are a large family of viruses and the subset of Coronaviridae, ranging from the common cold virus to more serious diseases such as SARS, MERS, and COVID-19 [5, 6].

Depending on the type of coronavirus, the symptoms can range from common cold symptoms to fever, cough, shortness of breath, and acute respiratory problems. The patient may also have a seemingly unexplained cough for several days. Unlike the SARS, the MERS-coronavirus (MERS-CoV) affects not only the respiratory system but also other vital organs in the body, such as the kidneys and the liver. In acute cases, gastrointestinal problems such as diarrhea, acute respiratory failure, blood coagulation disorders, and renal failure have also been reported, which may lead to the patient’s need for hemodialysis [5–7].

Some people have no symptoms or only mild symptoms. But in other people, COVID-19 can lead to serious problems such as pneumonia, not getting enough oxygen, and even death [6]. These symptoms are more common in people with other underlying problems, given that coronavirus symptoms and their effects on various organs are currently unknown and under investigation [6, 7].

There are several causes for the atraumatic splenic rupture (ASR):

Infectious causes, blood and metabolic diseases, pathophysiological disorders and iatrogenic conditions along with splenic rupture, Infectious mononucleosis (IM, mono) are the other causes of the atraumatic splenic rupture [4].

A patient with infectious mononucleosis and splenic enlargement (splenomegaly) should be closely monitored. The pathologic splenic rupture is the most common cause of death in patients with infectious mononucleosis [4]. It has been believed that increased intra-abdominal pressure or diaphragmatic contraction during severe coughing, vomiting, and defecation which leads to pressure on the spleen, is the cause of this type of splenic rupture [8]. However, Patel et al. believed that this was primarily the result of a progressive subcapsular hematoma that subsequently ruptured the capsule and caused hemoperitoneum [9]. Splenic rupture is traditionally treated with splenectomy. It is reasonable to avoid the possibility of sudden death as an early complication of splenic rupture and the risk of blood transfusions. Initially, the reconstruction of the spleen was considered as an option, but the difficulty in implementing this technique due to the enlargement and easy rupture of a sick spleen and the increased likelihood of rebleeding made it less desirable [9]. Based on the role of the spleen in immunity and overwhelming post-splenectomy infection (OPSI), there is a tendency to conservative management of splenic rupture in hemodynamically stable patients [9]. The survival rate of splenectomy in these patients in articles is close to 100% [10, 11]. Despite the potential death caused by the rupture of the spleen in infectious mononucleosis, the survival benefit is superior to the risk of sepsis due to post-splenectomy infection [10, 11].

Malaria is another cause of splenic rupture in malaria-prone areas. Most splenic rupture occurs in the acute phase of infection [12, 13]. In people undergoing hemodialysis and receiving heparin, non-traumatic rupture of the spleen has been reported because the edema of the spleen causes a hematoma under the splenic capsule. In people who are under treatment with thrombolytic drugs or taking anticoagulant drugs, a disturbance in the mechanism of homeostasis causes splenic rupture due to minor bumps [14, 15]. According to the above-mentioned discussions, can COVID-19 be another infectious cause of spontaneous splenic rupture (SSR)? Examining this issue requires further investigation and waiting for similar reports.

The evaluation of the spontaneous splenic rupture (SSR) in our case shows that this type of risk should also be considered in patients with COVID-19 who referred to medical centers with abdominal pain, and if more cases are reported, the correctness of this process can be commented on.

## Data Availability

All data generated or analysed during this study are included in this published article.

## Abbreviations

LUQ: Left Upper Quadrant
rtPCR: real-time Polymerase chain reaction
COVID-19: Coronavirus disease 2019
SSR: Spontaneous Splenic Rupture
ASR: Atraumatic Splenic Rupture
CT scan: Computed tomography scan
CBC: Complete Blood Count
BUN: Blood Urea Nitrogen
Cr: Creatinine
PT: Prothrombin Time
PTT: Partial Thromboplastin Time
INR: International Normalized Ratio
LFT: Liver Function Test
LDH: Lactate dehydrogenase
WBC: White Blood Cells
PLT: Platelets
HB: Hemoglobin
O2Sat: Oxygen Saturation
LE cell: Lupus Erythematosus cell
ANA: Anti-Nuclear Antibody
HIV: Human Immunodeficiency Virus
EBV: Epstein-Barr virus
IgM: Immunoglobulin M
IgG: Immunoglobulin G
VCA Antibody: Viral Capsid Antigen antibody
Cm: Centimeters
SARS: Severe Acute Respiratory Syndrome
MERS: Middle East Respiratory Syndrome
MERS-CoV: Middle East Respiratory Syndrome Coronavirus
IM: Infectious Mononucleosis
